# Space State Representation Corrections as an Aid in Pseudolite Positioning

**DOI:** 10.3390/s19194158

**Published:** 2019-09-25

**Authors:** Jacek Rapiński, Dariusz Tomaszewski

**Affiliations:** Institute of Geodesy, Faculty of Geodesy, Geospatial and Civil Engineering, University of Warmia and Mazury in Olsztyn, 10-719 Olsztyn, Poland; jacek.rapinski@uwm.edu.pl

**Keywords:** pseudolite, SSR, RTCM

## Abstract

In the presented study, the authors deal with the problem of transmission of pseudolite coordinates to the receiver. Nowadays, there is no uniquely specified method that would provide data about the position of the pseudolite to the GNSS receiver. There is also no technical standard that defines the explicit way of performing such transmission. Solutions presented in the literature are usually tailored to the described system, which is then suited to the specific situation. The article shows that the universal methods, involving the modification of transmitted broadcast ephemeris data, cannot be universally used. The modifications could not have been introduced due to the low resolution of the quantities that are transmitted in the ephemeris data, in relation to the values that would have to be sent by the pseudolite. To overcome the implementation problems, the authors propose two solutions. The first solution presented is the modification of the RTCM SSR frame. This approach allows replacing one of the existing satellites in space with the pseudolite, while the second method involves the use of new RTCM frame for sending the pseudolite position. Finally, a numerical example of the proposed solutions is presented. At the end of the manuscript, their advantages and implementation possibilities are discussed.

## 1. Introduction

The application of pseudolites for precise positioning is the subject of much research. There are many unresolved issues which must be addressed for pseudolites to become commercially available at a wide scale. One of the problems is how to send the correct pseudolite location to the receiver. An optimal solution would be to find a solution that does not require significant modification in the receiver firmware. Finding such a solution is difficult because modern receivers are able to calculate the positions of the satellites based on ephemeries data only. Regardless of the source (Internet, Broadcast data, and RTCM (Radio Technical Commission for Maritime) stream ) ephemeris data and its values are always the same. In recent years, no attempt has been made to definitively resolve the solution to this problem.

A universal solution to resolve this problem was made in 2012 [1], which describes the modification of orbital parameters of GNSS navigation data to make the receiver calculate constant pseudolite coordinates. The purpose of this modification was to transmit pseudolite position to the receiver without modifications in the receiver’s firmware or hardware. A similar idea was placed in a U.S. patent application [2]. From the mathematical point of view, it is possible and, thus, appropriate equations were introduced. This solution was used in further attempts to create system based on signals from pseudolites [3] and while creating the multi GNSS constellation described in [4]. An additional method was also used to simulate the creation of modern systems integrating GPS and pseudolite–GPS measurements [5]. A similar idea is presented in [6], but it assumes utilization of modified ephemeris data without using the standard broadcast GPS frames. This suggests that the receiver firmware has been appropriately and significantly modified. In the simulated system [7], the authors suggested the creation of separate frames for sending coordinates of both satellites and pseudolites. Older solutions described in the literature assume storing pseudolites positions in databases inside the mobile receiver or transmitting stored pseudolite positions via wireless networks [8,9].

Considering the universality criterion of the solution, the use of the algorithm presented in [1] seems to be an optimal solution. The method assumes that pseudolites position could be sent to the receiver using regular (satellites in space) ephemeries data. Therefore, the use of a pseudolite would not require any additional signals or data links, only the signal sent by the pseudolite itself. However, due to the construction of the frames, in which the broadcast data message is being sent, some implementation problems arise. Each data frame sent in a broadcast data message has a specified maximum and minimum size that can be transmitted. In the case of solutions presented in the above article, the problem arises at the time of sending the right value of right ascension of ascending node Ω. The idea was that this value must be time independent. In [1], $\dot{\Omega }$ is set to $72,921,151,467e^{-5}$ to match Earth’s rotation rate. The problem is that in the navigation message in subframe 5 word 5 is 16 bits for $\dot{\Omega }$ and 8 bits for SV (Satellite Vehicle) health [10]. The maximum value of $\dot{\Omega }$ is $2^{16}\times 2^{-43}$, which is much less than required, thus there is no possibility for $\dot{\Omega }-{\dot{\Omega }}_{e}$ to be 0 (equal). Therefore, it is not possible to implement the algorithm presented in the paper in a standard receiver, unless major modifications were to be made inside the receiver’s firmware. The method based on the modification of broadcast ephemeris data was abandoned because it was technically impossible to change broadcast ephemeris in described manner.

**Figure 1:**
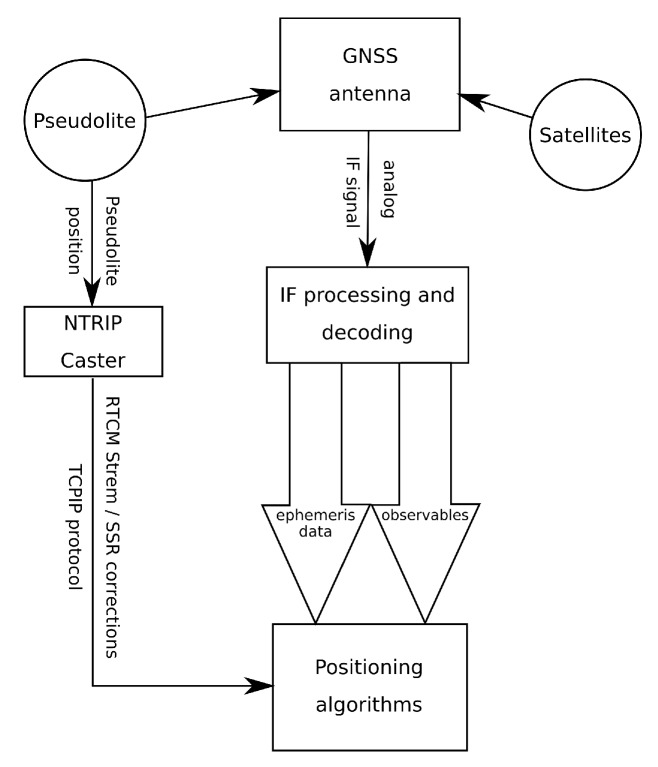
Concept of RTCM pseudolite corrections data flow.

To overcome the implementation problems, the authors propose two solutions. These solutions require access to the Internet and the use of external RTCM data streams, but are designed to minimize the system creator’s interference in receiver firmware. The first solution is based on the use of the RTCM 1060/1057 frame to send pseudolite SSR (Space State Representation) corrections data. The transmitted SSR data are recalculated in such a way that the receiver can reduce the current position of the satellite in space to the PL (pseudolite) position. The proposed algorithm requires small modification in the RTCM frame reading inside the receiver firmware. The second method involves the use of new RTCM frame to send the pseudolite position. This method is convenient but at the same time requires significant modification of the receiver firmware. Both solutions can be used regardless of the type of pseudolite and GNSS systems used in positioning. The concept of data flow in presented solution is depicted in Figure 1.

## 2. The Idea of Using SSR Corrections to Transmit Pseudolite Position

In modern precise GNSS positioning, apart from GNSS signals, auxiliary data are required. These data vary from technique to technique (for example, DGPS or RTK corrections, clock corrections, Earth orientation parameters, and antenna PVC and PVO) but are usually sent by TCP/IP protocol (Transmission Control Protocol/Internet Protocol) using NTRIP (Networked Transport of RTCM via Internet Protocol) caster/client in RTCM format. As one such type of auxiliary data, Space State Representation orbit corrections can be used to improve the precision of satellite positions calculated from broadcast ephemeris. One of the basic assumptions that led the developers of SSR corrections was to enable users of GNSS systems to perform PPP measurements in real time. Space State Representation corrections aim to minimize the errors of the GNSS control segment by providing satellite position and clock corrections with accuracy class of precise ephemeris. The use of SSR data enables increasing the accuracy of satellite position determination from 100 cm (broadcast ephemeries) to 5 cm [11]. These corrections are provided for example by IGS and are provided in messages 1057 (orbit corrections only) and 1060 (combined clock and orbit corrections). The IGS service declares that the accuracy of SSR products in similar to ultra-rapid half predicted orbit. The description of how to apply SSR orbit corrections can be found in RTCM Paper 142-2011-SC104-STD [12].

Corrected satellite coordinates $X_{orbit}$ (vector of coordinates) in Earth Centered Earth Fixed (*ECEF*) coordinate system are calculated by subtracting satellite position corrections δX from satellite position computed from broadcast ephemeris $X_{broadcast}$.

(1) $$X_{orbit}=X_{broadcast}-\delta X$$

Orbit corrections are defined in radial, along-track and cross-track directions. These values are contained in a δO vector. To obtain corrections in ECEF coordinate system δX, vector δO must be multiplied by matrix *E* containing radial, along and cross satellite unit vectors.

(2) $$E=\left[\begin{array}{ccc} e_{r}^{X} & e_{a}^{X} & e_{c}^{X}\\ e_{r}^{Y} & e_{a}^{Y} & e_{c}^{Y}\\ e_{r}^{Z} & e_{a}^{Z} & e_{c}^{Z} \end{array}\right]$$

Values of unit vectors $e_{r},e_{a},e_{c}$ are computed as:

(3) $$\begin{array}{c} e_{a}=\frac{\dot{r}}{|\dot{r}|} \end{array}$$

(4) $$\begin{array}{c} e_{c}=\frac{r\times \dot{r}}{|r\times \dot{r}|} \end{array}$$

(5) $$\begin{array}{c} e_{r}=e_{a}\times e_{c} \end{array}$$

where *r* and $\dot{r}$ are defined as satellite broadcast position vector and satellite broadcast velocity vector

(6) $$\begin{array}{c} r=X_{broadcast} \end{array}$$

(7) $$\begin{array}{c} \dot{r}={\dot{X}}_{broadcast} \end{array}$$

Orbit corrections vector consists of correction terms radial $\delta_{r}$, along-track $\delta_{a}$ and cross-track $\delta_{c}$ and its rates ${\dot{\delta }}_{r}$, ${\dot{\delta }}_{a}$, and ${\dot{\delta }}_{c}$ and can be described by Equation (8):

(8) $$\delta O=\delta O_{0}+\delta \dot{O}=\left[\begin{array}{c} \delta_{r}\\ \delta_{a}\\ \delta_{c} \end{array}\right]+\left[\begin{array}{c} {\dot{\delta }}_{r}\\ {\dot{\delta }}_{a}\\ {\dot{\delta }}_{c} \end{array}\right]\left(t-t_{0}\right)$$

where *t* refers to the current time and $t_{0}$ is reference time obtained from SSR Orbit Correction message. The final satellite position correction in ECEF δX is:

(9) δX=EδO

**Figure 2:**
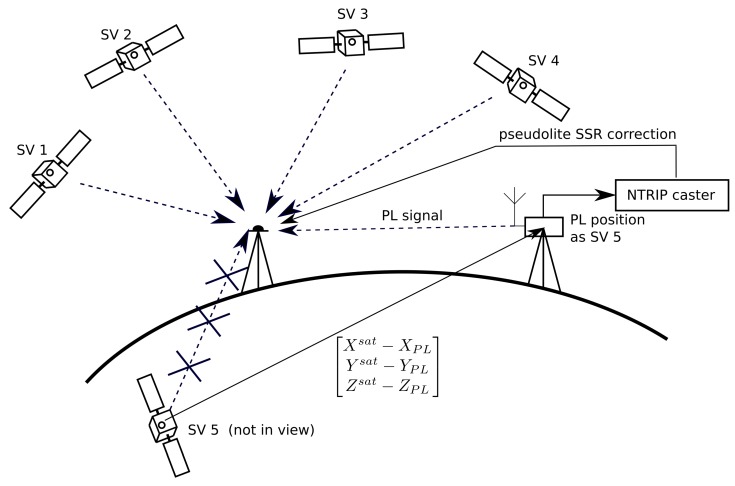
Idea of SSR satellite–pseudolite correction.

The main idea of the proposed solution is to choose the PRN number of existing satellite currently moving in space. Then, the pseudolite SSR correction is used to virtually move the position of the chosen satellite to the position of the pseudolite placed on the ground. According to the idea presented in [1] to identify pseudolite, one can use Pseudorandom Noise code number of the satellite that is currently not in view. Using this approach, additional observations are added to the measurement model. Thus, the pseudolite sends its signals pretending to be another satellite in space (Figure 2). Figure 2 shows the use of pseudolite during measurements. In this figure, pseudolite generates signal pretending to be satellite No. 5 (currently not in view). Then, based on the known actual position of satellite No. 5, a modified SSR correction is calculated. The final computed values are sent via the RTCM stream to the receiver.

The approach assumes manipulation of SSR corrections value ($\delta_{r}$, $\delta_{a}$, and $\delta_{c}$) and their rates (${\dot{\delta }}_{r}$, ${\dot{\delta }}_{a}$, and ${\dot{\delta }}_{c}$). The values of SSR vectors must be adjusted so that it would be possible to reduce the true satellite position to actual pseudolite position. Modification of Space State corrections should be made assuming that the values found in the unit radial, cross and along vectors ($e_{r},e_{a},e_{c}$) are determined for a satellite that is moving in space. That consequently leads to the transformations that are carried out on the actual *E* matrix. If the second term of Equation (1), δX, were equal to the satellite–pseudolite vector in each epoch, the resulting coordinates would be constant and equal to pseudolite position. Since matrix *E* depends on the satellite trajectory, only corrections δO can be modified. The satellite–pseudolite vector can be denoted as:

(10) $$\delta X=\left[\begin{array}{c} \Delta X\\ \Delta Y\\ \Delta Z \end{array}\right]=\left[\begin{array}{c} X^{sat}-X_{PL}\\ Y^{sat}-Y_{PL}\\ Z^{sat}-Z_{PL} \end{array}\right]+\left[\begin{array}{c} {\dot{X}}_{PL}^{sat}\\ {\dot{Y}}_{PL}^{sat}\\ {\dot{Z}}_{PL}^{sat} \end{array}\right]\left(t-t_{0}\right)$$

where $X^{sat},Y^{sat},Z^{sat}$ are satellite coordinates calculated from broadcast ephemeris at time $t_{0}$, $X^{PL},Y^{PL},Z^{PL}$ are pseudolite coordinates and ${\dot{X}}_{PL}^{sat}$, ${\dot{Y}}_{PL}^{sat}$, and ${\dot{Z}}_{PL}^{sat}$ are change rates of satellite–pseudolite coordinate vector from time $t_{0}$ to time *t*. From Equation (9), one can derive δO:

(11) $$\delta O=E^{-1}\delta X$$

which is equal to:

(12) $$\left[\begin{array}{c} \delta_{r}\\ \delta_{a}\\ \delta_{c} \end{array}\right]+\left[\begin{array}{c} {\dot{\delta }}_{r}\\ {\dot{\delta }}_{a}\\ {\dot{\delta }}_{c} \end{array}\right]\left(t-t_{0}\right)=E^{-1}\left[\begin{array}{c} X^{sat}-X_{PL}\\ Y^{sat}-Y_{PL}\\ Z^{sat}-Z_{PL} \end{array}\right]+E^{-1}\left[\begin{array}{c} {\dot{X}}_{PL}^{sat}\\ {\dot{Y}}_{PL}^{sat}\\ {\dot{Z}}_{PL}^{sat} \end{array}\right]\left(t-t_{0}\right)$$

when $t=t_{0}$, the second terms of Equation (12) are neglected, which yields satellite–pseudolite orbital corrections:

(13) $$\left[\begin{array}{c} \delta_{r}\\ \delta_{a}\\ \delta_{c} \end{array}\right]=E^{-1}\left[\begin{array}{c} X^{sat}-X_{PL}\\ Y^{sat}-Y_{PL}\\ Z^{sat}-Z_{PL} \end{array}\right]$$

The structure of SSR GPS orbit correction message is presented in Table 1 and Table 2 [13].

## 3. Application of Pseudolite SSR Corrections and New RTCM Message to Transmit Pseudolite Position

**Figure 3:**
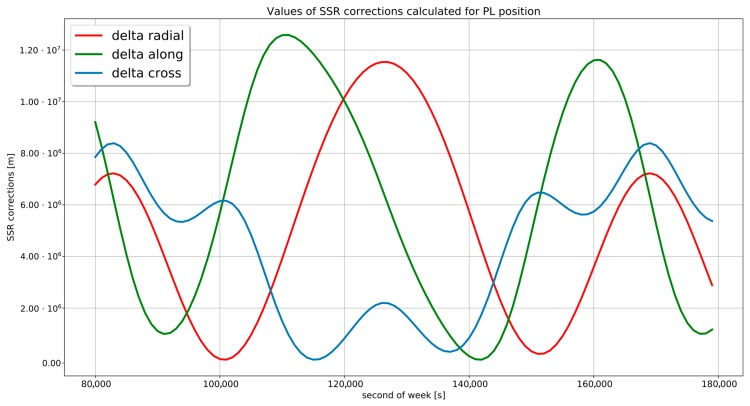
Minimized values of SSR corrections for pseudolite 24-h positioning session.

The use of unchanged resolution of SSR correction read is not possible because the maximum value that can be sent in $\delta_{a},\delta_{r},\delta_{c}$ fields is too small to send required increments. Resolution is defined as a value assigned to the less significant bit of received integer. the The sample values of pseudolite SSR corrections for the selected satellite during the 24-h measurement session are depicted in Figure 3. To present calculated Space State Representation in one graph, the smallest value has been subtracted from each quantities. Consequently, the actual value of the $\delta_{r}$ should be increased by the smallest distance between the satellite and the receiver. This results in a correction value of the order of $3.2\times 10^{7}$. This value significantly exceeds the available SSR data range shown in Table 2.

The above considerations have led to the conclusion that the problem of sending the pseudolite position cannot be solved without slight modification of the receiver’s firmware. In this article, the following two solutions are proposed.

### 3.1. Modification of Resolution and Processing Algorithm of SSR Corrections

As stated above, it is not possible to send standard RTCM 1057 SSR values appropriate for the pseudolite position. The assumed data range allows the maximum size of corrections ±209.7151 m and, in the considered case, the corrections should be around $3.2\times 10^{7}$ m. To increase the maximum data range, manipulations of two quantities may be considered. One can change the calculation’s resolution or the value of maximum sent integer (Table 2). Changing the maximum value sent in the stream (Max int) is problematic because it results from the number of bits defined in the RTCM frame. The simplest solution is to change the resolution in which the receivers firmware recalculates received SSR values. The proposed modification is shown in Table 3. The newly introduced resolution allows storing the values of $\delta_{r}$, $\delta_{a}$ and $\delta_{o}$ to an accuracy of 100 m. That, in return, increases the data range of these values to the order of $2.09\times 10^{8}$ for $\delta_{r}$ and $5.24\times 10^{8}$ for $\delta_{a}$ and $\delta_{o}$. To ensure accuracy of final SSR correction, the resolution of the SSR rates ${\dot{\delta }}_{r}$, ${\dot{\delta }}_{a}$ and ${\dot{\delta }}_{c}$ are modified as well (Table 3). The final correction value is sent with an accuracy of 0.0001 m. The introduced changes of satellite specific part of 1057 RTCM are marked in bold.

This solution is appropriate, assuming that the SSR message is provided every 1 s so that $(t-t_{0})=1$. Modification of the reading resolution allows for minimal modification in the receiver’s firmware; however, it requires continuous Internet communication during measurements.

### 3.2. New RTCM Message for Sending PL Location

As an alternative method, one can use dedicated RTCM frame to provide pseudolite position. As part of the RTCM standard, there are already defined frames for coordinate transmission. For example, the 1005/1006 RTCM frame is used for transmitting reference station coordinates [13]. The newly designed frame is created in a similar way (Table 4). This solution assumes expanding the receiver firmware with a module that allows reading a dedicated RTCM frame. Using dedicated RTCM frame allows one-time sending of PL coordinates, which in turn diminishes the bandwidth occupation problem.

The use of the author’s frame extends the possibility of sending a message regarding the position of the pseudolite. In data field 1 “Message number”, it is recommended to insert a value that has not been reserved in the RTCM specification [12]. Data field 2 “Pseudolite ID” is used to identify the device, the message refers to. Data field 3 “Coordinate system EPSG” allows entering the European Petroleum Survey Group (EPSG) number of the coordinate system, in which pseudolite coordinates are provided. Selection of the coordinate system extends the calculation capabilities of the receiver while using the designed RTCM message. This solution enables direct calculation in two- or three-dimensional local systems or in any nationally defined system without the need to perform coordinate transformation. Data field 4 “Provider ID” is used to identify the device that sends the message. In data field 5 “Ellipsoidal or Cartesian”, it is possible to determine whether the coordinates are in the ellipsoidal reference coordinate system or Cartesian coordinate system. Data fields 6–8 hold the pseudolite coordinates. Data contained in these fields depend on the coordinate system defined in data field 5. Cartesian rectangular coordinates are values of X, Y and Z distances measured from the center of the Earth specified at given reference system. It is also possible, within the ellipsoidal coordinate system, to use latitude (φ) and longitude (λ) along with ellipsoidal, orthogonal or normal altitude (*h*). Depending on the choice of coordinate system, the data fields reading resolution of submitted values will change. For the Cartesian system, the receiver’s firmware converts received integer values according to the predefined resolution of 0.001 m and at the same time in the case of an ellipsoidal system resolution value will be $1e^{-7}rad$. The set resolution values allow sending pseudolite coordinates with millimeter accuracy.

## 4. Numerical Example

The following section presents the case of using the discussed approaches to send pseudolite location data.The first approach requires the use of real ephemeris data to calculate the values found in the simulated RTCM 1060/1057 message. Satellite PRN14 was chosen for the exemplary calculation. Table 5 shows the ephemeris data for the selected epoch 93,600.0.

Using the ephemeris data, the actual coordinates of satellite are determined for the time of measurement $t_{0}$. On the basis of satellite coordinates and velocity matrix E containing unit radial, along and cross vectors are calculated.

(14) $$E=\left[\begin{array}{ccc} -0.4782192 & 0.10610773 & 0.87180706\\ -0.48431063 & 0.85995524 & -0.16099749\\ 0.73263197 & -0.49921752 & 0.46263621 \end{array}\right]$$

The actual coordinates of SV PRN 14 and true pseudolite coordinates are presented in Table 6. On the basis of established satellite and pseudolite position, in accordance with the idea of presented algorithm, the SSR correction vector was determined.

The developed values must be sent using a modified pseudolite RTCM message 1060/1057 according to the format specified in Table 3. A sample modified RTCM message is presented in Table 7. The abbreviation Int in the last column header stands for integer representation of the value.

An alternative to the method described above is to use dedicated RTCM message presented in the second approach. This solution does not require any computations, but only the transmission of properly encoded data using the described RTCM frame. For pseudolite coordinates contained in Table 6, the content of this message would be consistent with data in Table 8.

Proposed number selected in data field 1 is 4096 because it is the first RTCM message number not reserved in the specification [12]. Data fields 2 and 4 may hold any integer number denoting predefined provider IDs. Data field 3 contains the EPSG code of the pseudolite coordinates reference system [14]. In the example, it is 4326 which is the EPSG number of the WGS84 system. In the fifth field, boolean value indicate whether the coordinates found in data fields 6, 7 and 8 are ellipsoidal or Cartesian.

## 5. Conclusions

In this paper, the authors present two methods of providing pseudolite coordinates to the receiver. Both concepts are applicable and have some limitations. The first method is based on the modification of SSR orbital correction messages while the second one provides a proposition of new RTCM frame format for PL coordinates transmission. The first method is more difficult to implement on the transmitter side but requires only slight modification on the side of the positioning firmware. The second one requires implementation of a new RTCM message. Bandwidth occupation for a single message is similar (127 vs. 146 bits) but, since in the pseudolite SSR correction satellite position is constantly changing, corrections must be sent with 1-s interval. While using new RTCM message transmission, data are constant, thus it can be sent with much greater interval such as 1 min or more. Choosing between these two methods will mainly depend on the receiver that will be used for measurements. In some cases, applying the method using pseudolite SSR message will only require a few commands sent by the user to introduce necessary changes. However, with the possibility of greater interference in the receiver’s firmware, it is recommended to keep a fixed frame, in which it will be possible to send pseudolite coordinates.

## Figures and Tables

**Table 1:** Header part of RTCM 10,403.2 message 1057.

Symbol	Data Field	Number of Bits	Data Range	Resolution	Max Integer
No	Message number	12	-	-	-
$t_{0}$	GPS epoch time	20	0–604,799	1 s	524,287
Δt	SSR update interval	4	-	-	-
mmi	Multiple Message indicator	1	-	-	-
datum	Satellite reference datum	1	-	-	-
$t_{0}$	IOD SSR	4	-	-	-
prov	SSR Provider ID	16	-	-	-
sol	SSR Solution ID	4	-	-	-
*n*	No. of satellites	6	-	-	-

**Table 2:** Satellite specific part of RTCM 10,403.2 message 1057.

Symbol	Data Field	Number of Bits	Data Range	Resolution	Max Integer
ID	GPS Satellite ID	6	-	-	-
$\delta_{r}$	Delta radial	22	±209.7151	0.1mm	±2,097,151
$\delta_{a}$	Delta along-track	20	±209.7148	0.4 mm	±524,287
$\delta_{c}$	Delta cross-track	20	±209.7148	0.4 mm	±524,287
${\dot{\delta }}_{r}$	Delta radial rate	21	±1.048575	0.001 mm/s	±1,048,575
${\dot{\delta }}_{a}$	Delta along-track rate	19	±1.048572	0.004 mm/s	±262,144
${\dot{\delta }}_{c}$	Delta cross-track rate	19	±1.048572	0.004 mm/s	±262,144

**Table 3:** Modified satellite specific part of RTCM 10,403.2 message 1057.

Symbol	Data Field	Number of Bits	Data Range	Resolution	Max Integer
ID	GPS Satellite ID	6	-	-	-
$\delta_{r}$	Delta radial	22	±209,715,100.0	100 m	±2,097,151
$\delta_{a}$	Delta along-track	20	±52,428,700.0	100 m	±524,287
$\delta_{c}$	Delta cross-track	20	±52,428,700.0	100 m	±524,287
${\dot{\delta }}_{r}$	Delta radial rate	21	±104.8575	0.1 mm/s	±1,048,575
${\dot{\delta }}_{a}$	Delta along-track rate	19	±104.8572	0.4 mm/s	±262,144
${\dot{\delta }}_{c}$	Delta cross-track rate	19	±104.8572	0.4 mm/s	±262,144

**Table 4:** Pseudolite specific part of proposed RTCM message.

ID	Data Field	Data Range	Number of Bits	Resolution
1	Message number	uint	12	-
2	Pseudolite ID	uint	4	1
3	Coordinate system EPSG	uint	27	1
4	Privider ID	uint	16	1
5	Ellipsoidal or Cartesian	bool	1	1
6	Pseudolite X or φ	int32	32	0.001 m/$1e^{7}$ rad
7	Pseudolite Y or λ	int32	32	0.001 m/$1e^{7}$ rad
8	Pseudolite Z or *h*	int32	32	0.001 m

**Table 5:** Satellite PRN14 ephemeris data.

Parameter	Value
$\sqrt{a}$	5153.79589081
$t_{oe}$	93,600.0
$M_{0}$	1.05827953357
$e_{cc}$	0.00223578442819
Δn	$0.465376527657\cdot 10^{-8}$
ω	2.06374037770
$C_{us}$	$0.177137553692\cdot 10^{-5}$
$C_{uc}$	$0.457651913166\cdot 10^{-5}$
$C_{rs}$	88.6875
$C_{rc}$	344.96875
$C_{is}$	$-0.856816768646\cdot 10^{-7}$
$C_{ic}$	$0.651925802231\cdot 10^{-7}$
$i_{dot}$	$0.342514267094\cdot 10^{-9}$
$\Omega_{0}$	1.64046615454
$\dot{\Omega }$	$-0.856928551657\cdot 10^{-8}$
$i_{0}$	0.961685061380

**Table 6:** SV and PL coordinates/Vector of pseudolite SSR corrections.

	X	Y	Z
SV coordinates	−12,673,915.048	−12,833,858.558	19,416,961.501
PL coordinates	3,538,856.756	1,324,402.322	5,121,378.163
SSR correction	radial	along	cross
δ	−25,083,600.0	−3,318,500.0	5,241,300.0
$\dot{\delta }$	−56.5011	−64.6598	9.8419

**Table 7:** Sample modified satellite specific part of RTCM 10,403.2 message 1057.

Symbol	Data Field	Number of Bits	Value	Resolution	Integer
ID	GPS Satellite ID	6	14	-	14
$\delta_{r}$	Delta radial	22	−25,083,600.0	100 m	−250,836
$\delta_{a}$	Delta along-track	20	−3,318,500.0	100 m	−33,185
$\delta_{c}$	Delta cross-track	20	5,241,300.0	100 m	52,413
${\dot{\delta }}_{r}$	Delta radial rate	21	−56.5011	0.1 mm/s	−565,011
${\dot{\delta }}_{a}$	Delta along-track rate	19	−64.6598	0.4 mm/s	−161,649
${\dot{\delta }}_{c}$	Delta cross-track rate	19	9.8419	0.4 mm/s	24,605
	sum:	127			

**Table 8:** Sample pseudolite specific part of designed RTCM message.

ID	Data Field	Data Format	Number of Bits	Resolution	Message
1	Message number	uint	12	-	4096
2	Pseudolite ID	uint	5	1	1
3	Coordinate system EPSG	uint	27	1	4326
4	Provider ID	uint	5	1	1
5	Ellipsoidal or Cartesian	bool	1	1	1
6	Pseudolite X	int32	32	0.01 m	353,885,675
7	Pseudolite Y	int32	32	0.01 m	132,440,232
8	Pseudolite Z	int32	32	0.01 m	512,137,816
		sum:	146		
